# Distinct clinical clusters of paediatric patients with status epilepticus: Retrospective cohort study

**DOI:** 10.1111/dmcn.70184

**Published:** 2026-01-30

**Authors:** Richard J Burman, Anne Steinberg, Jamie Norris, Giorgio Selmin, Tommaso Fedele, Richard E Rosch, Georgia Ramantani

**Affiliations:** ^1^ Department of Pediatric Neurology University Children's Hospital Zurich and University of Zurich Zurich Switzerland; ^2^ UCL Institute of Health Informatics London UK; ^3^ Institute of Cognitive Neuroscience National Research University Higher School of Economics Moscow Russian Federation; ^4^ Department of Basic and Clinical Neuroscience, Institute of Psychiatry, Psychology and Neuroscience King's College London UK

## Abstract

**Aim:**

To characterize the clinical features, management, and outcomes of paediatric patients with status epilepticus, and to explore whether distinct clinical subgroups can be identified from clinical descriptions.

**Method:**

This was an exploratory retrospective single‐centre cohort study of paediatric status epilepticus admissions to Switzerland's largest tertiary‐level paediatric hospital. We analysed 642 status epilepticus admissions from 467 patients (230 females; median age at first 4 years 11 months [interquartile range 1 year 5 months–7 years 5 months]). We applied descriptive statistics and machine‐learning approaches. A *k*‐means clustering algorithm was used to identify distinct clinical subgroups, while least absolute shrinkage and selection operator regression tested whether clinical metrics could predict mortality.

**Results:**

Age‐related differences in status epilepticus aetiology were observed: infants and younger children more often presented with acute symptomatic causes, whereas older children and adolescents were more likely to have pre‐existing epilepsy. Out‐of‐hospital treatment was associated with faster treatment initiation and better treatment response. Shorter status epilepticus onset to treatment latency correlated with higher response rates and reduced need for intensive care. Cluster analysis identified three clinical subgroups: (1) younger patients with acute status epilepticus associated with an infection, including febrile status epilepticus (‘febrile seizure’ cluster); (2) younger patients with acute status epilepticus and a more severe in‐hospital course (‘para‐infectious’ cluster); and (3) older patients with an established epilepsy diagnosis (‘known epilepsy’ cluster). Across the cohort, progressive epilepsy aetiology and a previous diagnosis of epilepsy were associated with increased mortality risk.

**Interpretation:**

Paediatric status epilepticus comprises clinically distinct subgroups that are identifiable from routine clinical data. Such data‐driven clinical clustering may help refine risk stratification and inform clinical decision making in paediatric status epilepticus.

## Graphical abstract

Large Swiss cohort of paediatric status epilepticus demonstrates that treatment delay (> 60 min) directly contributes to morbidity. Machine‐learning analysis of routine clinical data identifies three distinct patient clusters: febrile status epilepticus, severe para‐infectious status epilepticus, and status epilepticus in children with known epilepsy.

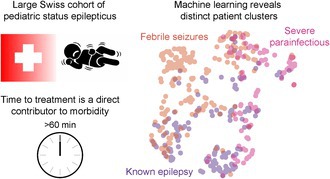

**Abbreviations**
ICU: intensive care unit
LASSO: least absolute shrinkage and selection operator
NCSE: non‐convulsive status epilepticus

**What this paper adds**

- Younger patients commonly present with status epilepticus due to an acute aetiology whereas older patients often have known epilepsy.
- Early out‐of‐hospital treatment is associated with improved response to first‐line medication.
- Treatment delays longer than 60 minutes are associated with an increased need for intensive care and second‐line therapy.
- Cluster analysis identifies three status epilepticus subgroups with distinct clinical characteristics.
- Mortality is higher in progressive status epilepticus and lower in infection‐related cases of status epilepticus.

Status epilepticus is a severely abnormal brain state characterized by prolonged, unrelenting seizures. Status epilepticus remains the most common neurological emergency in children, with this vulnerable population being at risk of significant short‐ and long‐term morbidity.^1^, ^2^, ^3^ However, predictors of poor outcomes vary widely across studies,^4^, ^5^, ^6^ probably reflecting heterogeneities in patients’ cohorts and healthcare settings.

Status epilepticus occurs both in paediatric patients with pre‐existing epilepsy or other neurodevelopmental conditions and in children with previously typical development, particularly in the context of febrile seizures.^7^, ^8^ Although the underlying aetiology significantly affects both the immediate treatment and longer‐term outcomes,^9^, ^10^ it remains unclear which clinical features at initial presentation best stratify patients.^11^ Furthermore, status epilepticus aetiologies and outcomes are affected by regional disease burden and healthcare infrastructure. For example, acute symptomatic causes, such as intracranial infections, are a leading cause of status epilepticus in low‐ and middle‐income countries,^12^, ^13^ while in high‐income countries status epilepticus is often caused by remote genetic and structural abnormalities.^14^

One of the major challenges in managing paediatric status epilepticus is dealing with the large heterogeneity of clinical presentations. The aetiology, semiology, and duration vary widely, yet the treatment follows the same algorithm. For example, response to first‐line treatment with benzodiazepines differs on the basis of latency from status epilepticus onset to time to treatment initiation, while the clinical presentation of status epilepticus and failure to respond to benzodiazepines are associated with poorer outcomes.^15^, ^16^, ^17^, ^18^ Identifying patterns in clinical features that predict response could help rationalize care in paediatric status epilepticus.^19^

Similarly, another important clinical pursuit has been trying to identify clinical parameters that may predict outcomes, notably mortality, of children with status epilepticus. Previous work has implicated variables such as clinical and semiological features of the patient during status epilepticus,^20^ prehospital treatment, and refractoriness to treatment^21^ as important predictors of mortality. However, a key limitation of these predictions is that the statistical tests used are often impeded by the variance from the heterogeneity in paediatric patients with status epilepticus.^9^ It is here that using novel machine‐learning approaches may prove useful in managing the diversity of clinical presentations and identifying clinically relevant trends.^22^

We report the clinical features of 642 episodes of status epilepticus from 467 patients presenting to the largest tertiary children's hospital in Switzerland. Using machine learning, we delineated distinct clusters of patients on the basis of clinical features at presentation and during admission. These findings provide insights into the heterogeneity of paediatric status epilepticus and highlight characteristics unique to Swiss health care.

## METHOD

### Study design

This retrospective cohort study was conducted at the largest tertiary paediatric hospital in Switzerland, including data from 2010 to 2022. The study received approval from the local ethics committee (Kantonale Ethikkommission Zürich, KEK‐ZH PB‐2020‐02580) for the retrospective use of anonymized, routinely collected clinical data. Owing to the retrospective nature of this study, patients’ consent was not required.

### Selection of patients

Patients with a clinical diagnosis of status epilepticus were included in the study. Status epilepticus was defined as a prolonged clinical seizure lasting longer than 5 minutes or recurrent seizures lasting longer than 30 minutes without interictal return to baseline. Since there is no universally accepted minimum duration for a definition of non‐convulsive status epilepticus (NCSE) without prominent motor features, the same temporal cut‐offs were applied in this cohort. However, given the nature of NCSE presentations, the precise onset is not usually clearly defined, and recognition typically occurs well after the longer than 5‐minute cut‐off. Cases were identified through a retrospective review of emergency department admission codes and electroencephalography (EEG) reports, and diagnoses were confirmed by an epileptologist (GR) following an individual review of patients’ records. Treatment followed the local status epilepticus protocol, which includes first‐line treatment with a short‐acting benzodiazepine, namely diazepam, midazolam, or lorazepam, depending on availability and administration routes. Other treatments were classified as second‐line treatments for this study. Patients younger than 1 month (neonates) and those with electrical status epilepticus in sleep were excluded from the study.

### Clinical variables

Data collected included age, sex, previous and acute neurological diagnoses, status epilepticus aetiology and semiology, status epilepticus duration, intensive care unit (ICU) admission, and antiseizure treatments received. The aetiology of pre‐existing epilepsy was defined by the latest International League Against Epilepsy framework.^23^ Status epilepticus semiology and aetiology were defined according to the latest International League Against Epilepsy multi‐axial status epilepticus classification.^24^ Prolonged seizures lasting longer than 5 minutes were classified as ‘continuous’ convulsive status epilepticus while recurrent seizures presenting as multiple discrete episodes without cognitive recovery were classified as ‘intermittent’ status epilepticus. We excluded axis 3, ‘electroencephalographic correlates’, because EEG was not routinely performed for all patients presenting with status epilepticus and was largely used to diagnose episodes of NCSE. For semiology (axis 1), patients were grouped by the presence or absence of prominent motor symptoms, then further classified as generalized or focal. For aetiology (axis 2), categories included ‘acute’ for status epilepticus caused by acute systemic illness or neurological injury (e.g. infection), ‘remote’ for status epilepticus following previous neurological injury (e.g. post‐stroke), ‘progressive’ for status epilepticus resulting from extending neurological disease (e.g. brain tumour), ‘syndrome’ for status epilepticus presenting as part of a defined electroclinical syndrome (e.g. Dravet syndrome), and ‘unknown’ if the underlying aetiology of status epilepticus was not found during admission. Febrile status epilepticus was defined as status epilepticus provoked by hyperthermia (>38.4 °C) without previous afebrile seizures or acute neurological disease.^25^ Response to treatment was assessed by the level of intervention required to terminate the status epilepticus clinically. Patients unresponsive to first‐line treatment with multiple doses of short‐acting benzodiazepines were classified as benzodiazepine‐resistant. Mortality was determined by reviewing hospital records, including any deaths reported up to 2024.

### Data collection and statistical analysis

Data collection and management was conducted using the REDCap (Research Electronic Data Capture) Web‐based platform.^26^ To ensure consistency during review of medical records, data extraction was standardized using a pre‐defined abstraction form with explicit variable definitions. The study team jointly reviewed a subset of records before full cohort extraction to calibrate interpretation and application of variables. Ambiguous cases were discussed contemporaneously among investigators to achieve consensus. This process was intended to minimize variability in data capture and maintain consistency across reviewers. Formal statistics for interrater reliability were not calculated. Continuous variables were summarized as medians with interquartile ranges (IQRs) and categorical variables as counts with percentages. Predictors of benzodiazepine treatment failure were assessed using odds ratios (OR) and 95% confidence intervals (CIs). Features associated with treatment response were determined by investigating associations with key clinical variables including place and timing of treatment, ICU admission, and mortality.

### Cluster analysis

To identify subgroups of patients with distinct clinical profiles, we performed unsupervised clustering using *k*‐prototypes,^27^ an extension of *k*‐means clustering that handles both numerical and categorical data. Clustering was performed at three stages of the clinical course: at presentation to our facility, during admission, and using all available data after admission. Continuous variables were standardized to have zero mean and unit variance before clustering. The *k*‐prototypes were fitted for *k* clusters, with *k* ranging from 1 to 6, and the associated cost value for each *k* was recorded. The elbow method was then used to determine the optimal number of clusters on the basis of these cost values. This process identified the optimal number of clusters and produced a cluster index for each patient at each stage. No additional manual weighting was applied between numerical and categorical variables, and their relative contribution followed the default formulation for *k*‐prototypes. Cluster stability was assessed using a pairwise co‐assignment‐based robustness metric, quantifying the consistency of membership in patient clusters across repeated model fits under different random initializations.^28^ Following clustering, factor analysis of mixed data was applied to the data at each stage. Factor analysis of mixed data, a variant of principal component analysis that accommodates both numerical and categorical data, allows plotting of individual patients as points, colour‐coded according to their cluster assignment.

### Mortality prediction

Patients with reported mortality during paediatric hospital follow‐up were analysed to identify predictors of mortality. Using all available clinical features for each patient, including data from their first status epilepticus episode, we used a least absolute shrinkage and selection operator (LASSO) regression model to identify significant predictors. Age was stratified as younger than 5 years or at least 5 years, consistent with the diagnostic criteria for febrile seizures. We optimized the penalty parameter *λ* using 10‐fold cross‐validation to minimize deviance. We then used these selected predictors in a logistic regression model to predict mortality using only the identified subset of clinical variables. Model performance was assessed using a receiver operator curve. We report odds ratios and their 95% CIs for each predictor. Missing data were partly imputed: ‘unknown’ was treated as a category in categorical variables and data sets with more than six of these were removed; missing numerical values were mean‐imputed.

## RESULTS

**FIGURE 1:**
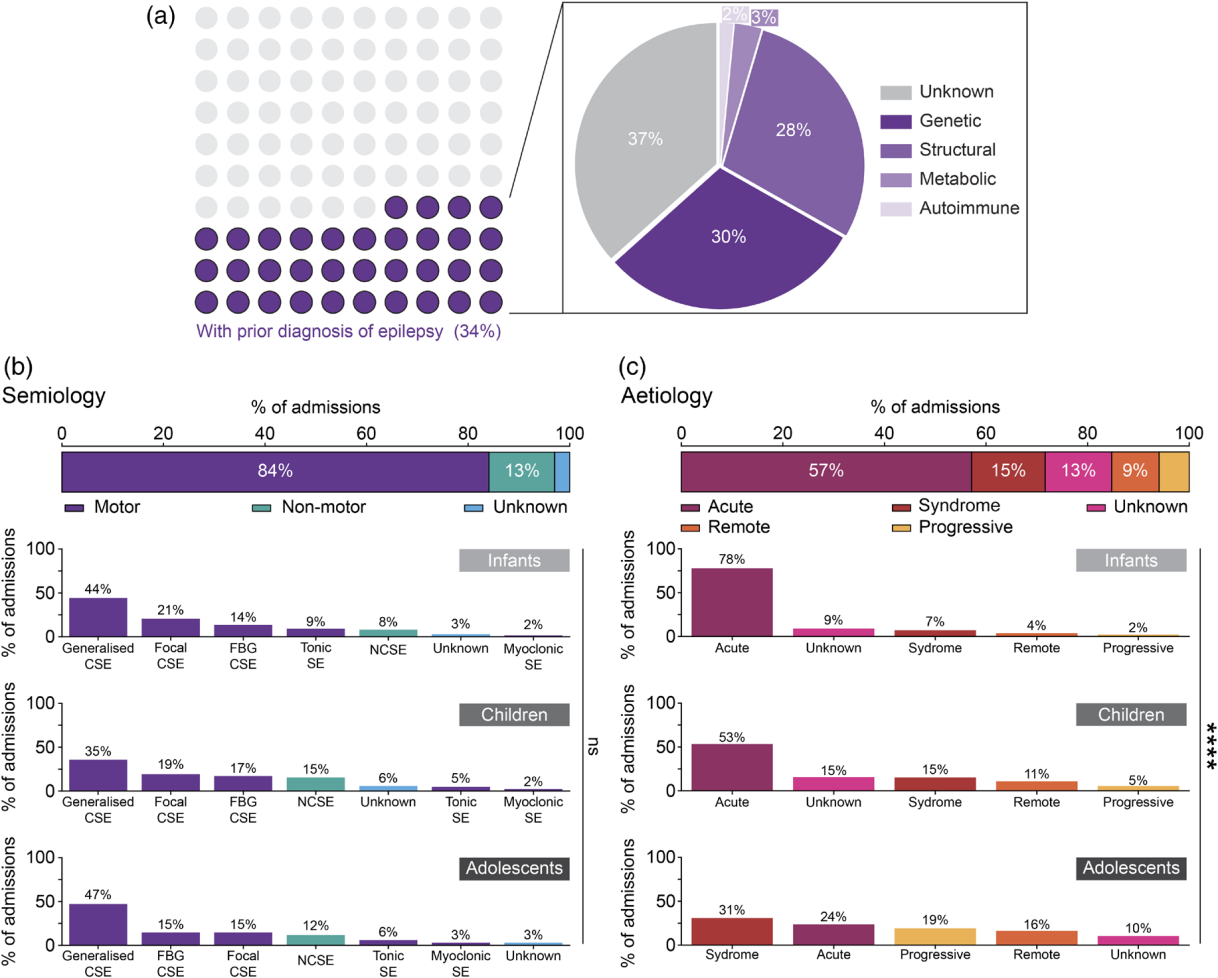
Overview of patients in the cohort, highlighting the distribution of semiology and aetiology across episodes of paediatric status epilepticus for the full cohort and according to distinct age groups. (a) Overview plot showing the distribution of patients with (purple) compared with those without (grey) pre‐existing epilepsy. The pie chart details the distribution of diagnoses among patients with pre‐existing epilepsy (ordered by the percentage of patients in each category). (b) Stacked bar plot showing the percentage of patients presenting with status epilepticus with prominent motor manifestation compared with those without. Below, bar plots show the distribution of specific semiologies stratified by age group (infants, children, and adolescents). Across age groups, no significant differences were observed in the distribution of semiologies (*χ* ^2^ statistic = 18.2, degrees of freedom 12, *p* = 0.11, *χ* ^2^ test). (c) Stacked bar plot showing the breakdown of different aetiologies underlying episodes of status epilepticus in this cohort. A significant difference in aetiologies was observed across age groups (*χ* ^2^ statistic = 87.4, degrees of freedom 8, *p* < 0.001). ****p < 0.001. Abbreviations: CSE, convulsive status epilepticus; FBG, focal to bilateral generalized convulsive status epilepticus; NCSE; non‐convulsive status epilepticus; ns, non‐significant; ‘Syndrome’, electroclinical syndrome.

Our cohort consisted of 467 patients (49% female, 230 out of 467). The median age at first presentation with status epilepticus was 4 years 11 months (IQR 1 year 5 months–7 years 5 months). Among these, 34% (159 out of 467) had a previous diagnosis of epilepsy, with genetic aetiology accounting for most (30%, 48 out of 159) of these cases (Figure 1a).

From this cohort, we analysed 642 episodes of status epilepticus (Table 1). The median age at admission was 4 years 6 months (IQR 1 year 8 months–7 years 10 months). Most patients (82%, 383 out of 467) had a single status epilepticus admission, with most episodes (58%, 370 out of 642) presenting in continuous status epilepticus according to clinical and/or EEG records. In most episodes of status epilepticus (63%, 297 out of 471 in episodes where time was known), the latency from status epilepticus onset to admission exceeded 1 hour. While the time to first treatment was poorly documented (i.e. unavailable for 31% of status epilepticus episodes, 200 out of 642), among episodes with recorded times (*n* = 442), patients received their first antiseizure medication within 1 hour of status epilepticus onset 79% (348 out of 442) of the time. Most episodes of status epilepticus (61%, 389 out of 642) had treatment initiated outside the hospital. Status epilepticus lasted longer than 1 hour in 49% (239 out of 488) of episodes for which these data were available (*n* = 488). More than half of the status epilepticus episodes (51%, 324 out of 642) required ICU admission, with 35% (116 out of 324) of these cases requiring intubation. During most episodes (57%, 367 out of 642), treatment beyond first‐line short‐acting benzodiazepines was required.

**TABLE 1:** Clinical and treatment profile of status epilepticus episodes.

Median (IQR) age at admission (years:months, *n* = 467 patients)	4:6 (1:8–7:10)
Number of admissions per patient (*n* = 467 patients)	
1	383 (82%)
2	53 (11%)
3	14 (3%)
≥ 4	15 (3%)
Type of status epilepticus (*n* = 642 episodes)	
Continuous	370 (58%)
Intermittent	272 (30%)
Unreported	80 (12%)
Duration of status epilepticus before admission (*n* = 642 episodes)	
< 10 minutes	51 (8%)
10–30 minutes	39 (6%)
31–60 minutes	84 (13%)
> 60 minutes	297 (46%)
Unreported	171 (27%)
Time to first‐line treatment (*n* = 642 episodes)	
< 10 minutes	155 (24%)
10–30 minutes	130 (20%)
31–60 minutes	63 (10%)
> 60 minutes	82 (13%)
Self‐limiting	12 (2%)
Unreported	200 (31%)
Place of first treatment (*n* = 642 episodes)	
In‐hospital	197 (31%)
Out‐of‐hospital	389 (61%)
No treatment required^a^	12 (2%)
Unknown	44 (7%)
Total duration of status epilepticus (*n* = 642 episodes)	
< 10 minutes	2 (1%)
10–30 minutes	78 (12%)
30–60 minutes	169 (26%)
> 60 minutes	239 (37%)
Unknown	154 (23%)
Intensive care required (*n* = 642 episodes)	
Admission to PICU	324 (51%)
Intubation^b^	116 (35%)
Level of intervention required (*n* = 642 episodes)	
First‐line treatment only	263 (41%)
More than first‐line treatment	367 (57%)
No treatment	12 (2%)

^a^ Self‐limiting refers to an episode of status epilepticus that resolved without the need for antiseizure medications.

^b^ Of those admitted to the PICU.

Abbreviations: IQR, interquartile range; PICU, paediatric intensive care unit.

In our cohort, 84% (538 out of 642) of status epilepticus episodes were characterized by prominent motor features (convulsive), while only 13% (83 out of 642) consisted of seizures without apparent motor manifestations (non‐convulsive; Figure 1b). Status epilepticus semiology was largely consistent across age groups (*p* = 0.11), with generalized convulsive status epilepticus being the most common. Acute brain insults accounted for 57% of status epilepticus episodes, with significant age‐related variation (Figure 1c; *p* < 0.001). Acute aetiologies were most common in infants (78%, 144 out of 185) and children (53%, 207 out of 389), whereas adolescents were more likely to have status epilepticus associated with an underlying electroclinical syndrome (31%, 21 out of 68).

**FIGURE 2:**
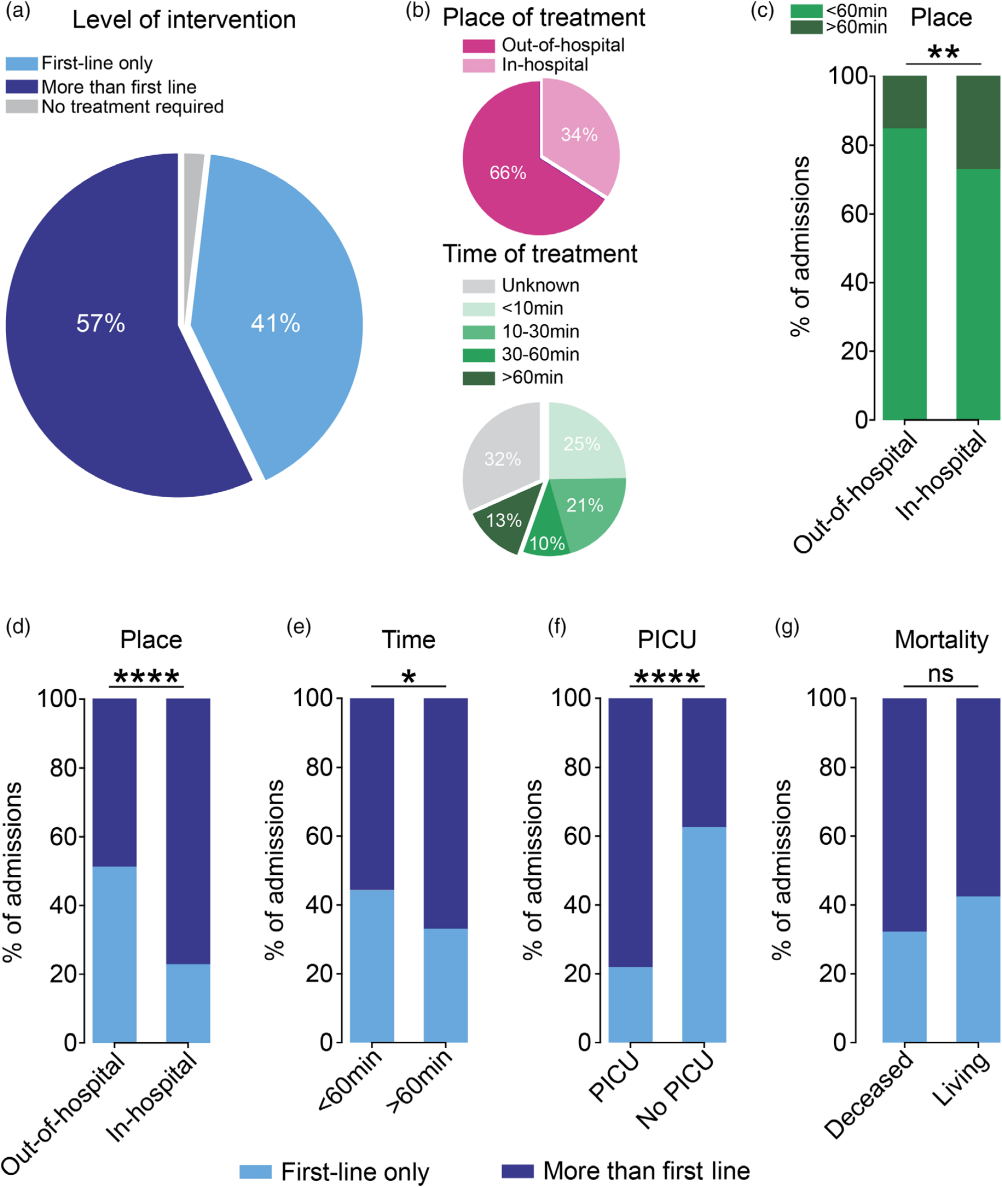
Timing and location of first‐line treatment are associated with treatment response. (a) Breakdown of status epilepticus episodes responding to first‐line treatment compared with those requiring additional intervention. Patients who did not require treatment to terminate their status epilepticus were excluded from subsequent analysis. (b) Location (top) of first‐line treatment administration and latency from status epilepticus onset to treatment initiation (bottom). (c) Patients who received their first treatment in‐hospital were significantly more likely to receive treatment more than 60 minutes after seizure onset (out‐of‐hospital <60 minutes 85% and > 60 minutes 15% vs in‐hospital <60 minutes 73% and > 60 minutes 27%; odds ratio (OR) 2.17, 95% confidence interval (CI) 1.3–3.5, *p* = 0.003, Fisher's exact test). (d) Patients who received their first dose in‐hospital were significantly more likely not to respond to first‐line treatment (out‐of‐hospital first‐line only 51% and more than first line 49% vs in‐hospital first‐line only 23% and more than first line 77%; OR 3.4, 95% CI 2.3–4.9, *p* < 0.001, Fisher's exact test). (e) Delays of more than 60 minutes to first treatment were associated with a higher likelihood of requiring treatment beyond the first line (<60 minutes first‐line only 45% and more than first line 55% vs >60 minutes first‐line only 33% and more than first line 67%; OR 1.6, 95% CI 1.0–2.8, *p* = 0.04, Fisher's exact test). (f) Patients who required treatment beyond the first‐line treatment were significantly more likely to require admission to the PICU (first‐line only 22% and more than first line 78% vs no PICU first‐line only 62% and more than first line 38%; OR 5.7, 95% CI 4.0–8.1, *p* < 0.001, Fisher's exact test). (g) Response to first‐line treatment was not associated with patients’ mortality (deceased first‐line only 32% and more than first line 68% vs living first‐line only 42% and more than first line 58%; OR 1.2, 95% CI 0.6–2.2, *p* = 0.75, Fisher's exact test). Abbreviation: PICU, paediatric intensive care unit.

Previous studies have demonstrated that delayed treatment, particularly when the duration of status epilepticus exceeds 60 minutes, is linked to worse outcomes.^15^, ^16^, ^17^ To evaluate this association in our cohort, we analysed the timing of treatment initiation. Among the 98% (630 out of 642) of episodes in which antiseizure medication use was documented (Figure 2a), 62% (389 out of 630) received their first medication out‐of‐hospital (Figure 2b). For cases with recorded times and requiring antiseizure medications, 36% (155 out of 442) received antiseizure medication within 10 minutes of status epilepticus onset. Patients treated in‐hospital were significantly more likely (OR 2.17, 95% CI 1.3–3.5, *p* = 0.003, Fisher's exact test) to receive their first treatment more than 1 hour after status epilepticus onset (Figure 2c).

Comparing patients who responded to first‐line treatment with those requiring higher levels of intervention, we found that in‐hospital first‐line treatment was associated with a greater likelihood of requiring higher levels of intervention (Figure 2d; OR 3.4, 95% CI 2.3–4.9, *p* < 0.001, Fisher's exact test). In addition, delays to first treatment longer than 60 minutes were associated with increased need for advanced care (Figure 2e; OR 1.6, 95% CI 1.0–2.8, *p* = 0.04, Fisher's exact test) and ICU admission (Figure 2f; OR 5.7, 95% CI 4.0–8.1, *p* < 0.001, Fisher's exact test). However, no relation was found between response to first‐line treatment and mortality (Figure 2g; OR 1.2, 95% CI 0.6–2.2, *p* = 0.75, Fisher's exact test). All odds ratio values are unadjusted and reflect exploratory analysis.

**FIGURE 3:**
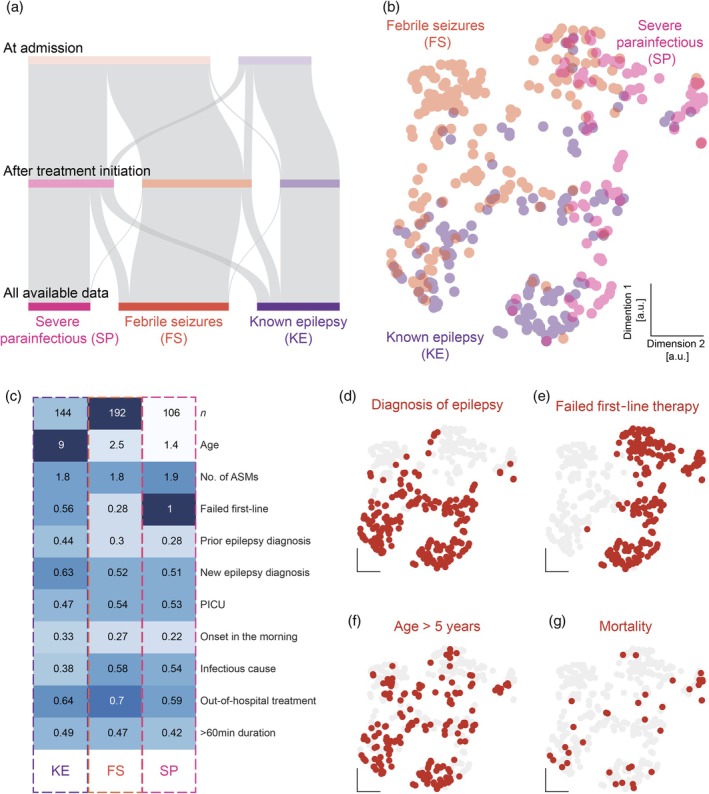
Clinical subgroup identification through clustering analysis. (a) Sankey diagram showing clustering results at three stages during the clinical course: (1) at admission, (2) after treatment initiation, and (3) using all available data of patients (excluding mortality). The width of the grey bars indicates relative numbers of patients, with connections across time indicating crossover between clusters. (b) Two‐dimensional UMAP embedding of all clinical data, colour‐coded by final cluster assignment. (c) Heat map displaying within‐cluster averages of the 10 clinical features contributing most significantly to final clustering. (d–f) UMAP plots highlighting subgroups in red: (d) patients with a diagnosis of epilepsy at any point; (e) patients who failed first‐line therapy; (f) patients older than 5 years; (g) patients who passed away at any point during follow‐up. Abbreviations: ASMs, anti‐seizure medications; FS, febrile seizure cluster; KE, known epilepsy cluster; PICU, paediatric intensive care unit; SP, severe parainfectious cluster; UMAP, Uniform Manifold Approximation and Projection.

Using unsupervised *k*‐prototypes clustering, we identified two main groups at admission: younger patients with acute aetiologies, and older patients with heterogeneous aetiologies (Figure 3a). These groups were further subdivided into (1) younger patients with acute status epilepticus in the context of infection including febrile status epilepticus (‘febrile seizure’ cluster), (2) younger patients with acute status epilepticus with more severe in‐hospital courses (‘para‐infectious’ cluster), and (3) older patients with pre‐existing epilepsy (‘known epilepsy’ cluster) (Figure 3b,c). Note that these cluster labels represent post‐hoc summary descriptions of the in‐cluster characteristics of patients. There is significant overlap, and individual patients, for example those with ‘febrile seizures’ as a clinical diagnosis, may still more closely align by clinical features with one of the other clusters; and patients in the ‘febrile seizure’ cluster may not meet clinical diagnostic criteria for febrile seizures. We also analysed the distribution of key clinical parameters across these three clusters. While a previous diagnosis of epilepsy and failure to respond to the first‐line treatment were concentrated in specific groups of patients (Figure 3d,e), age (notably >5 years) and mortality showed a more scattered distribution across the cohort (Figure 3f,g). Robustness scores were 0.986 at admission, 0.875 during admission, and 0.691 when using all available post‐admission data.

**FIGURE 4:**
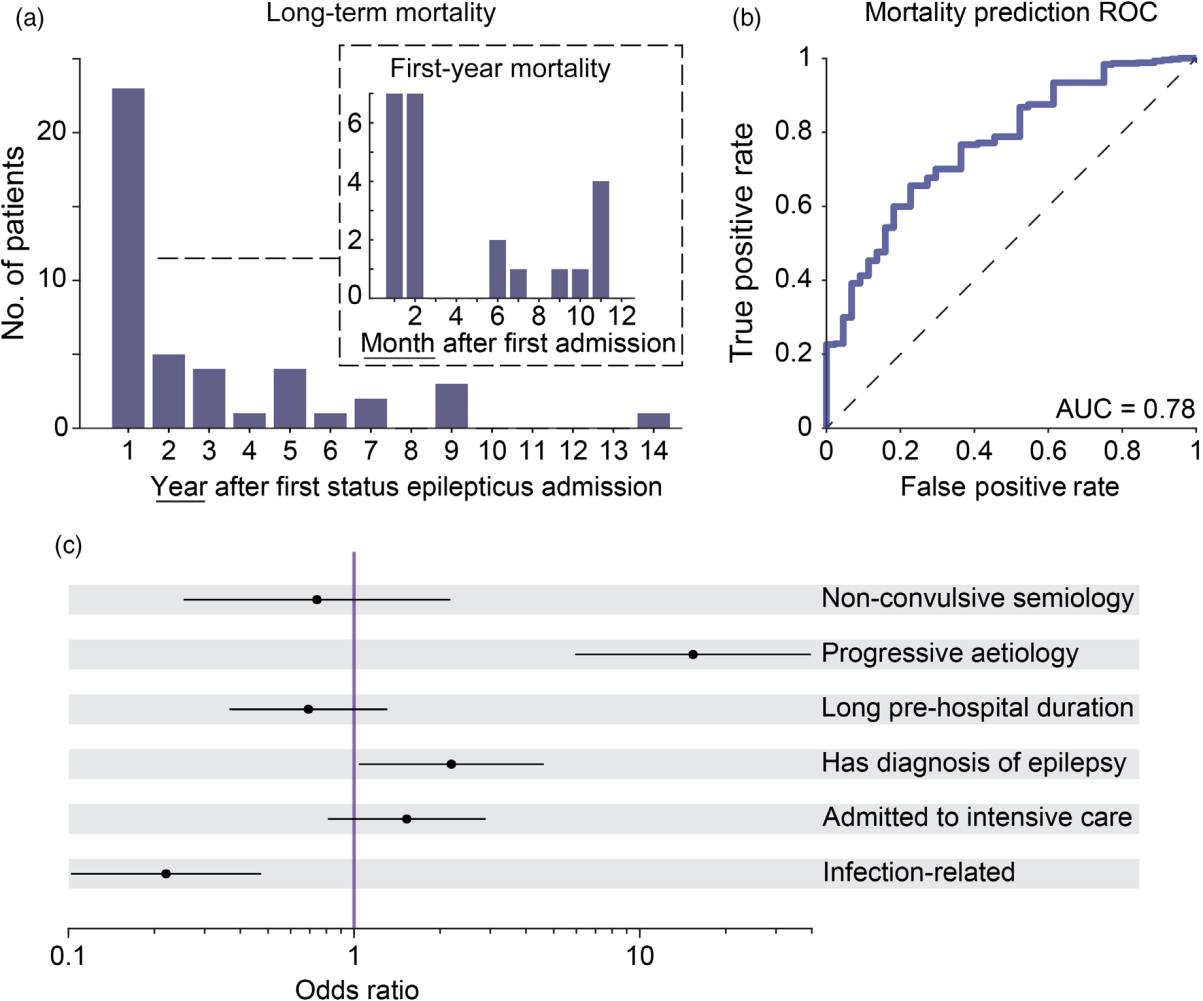
Predictive factors of mortality after paediatric status epilepticus. (a) Histogram of long‐term mortality, and (inset) first‐year mortality after first hospital admission for status epilepticus. (b) Performance of a logistic regression model on the basis of clinical predictors of mortality identified through LASSO regression. The model achieves an area under the curve of 0.78, indicating good predictive accuracy. (c) Odds ratios (OR) and 95% confidence intervals (CIs) for individual clinical predictors contributing to the logistic regression model. Individual values for OR (95% CIs), *p*‐values: ‘Non‐convulsive semiology’ OR = 0.74 (0.25–2.17), *p* = 0.6; ‘Progressive aetiology’ OR = 15.37 (5.95–39.73), *p* < 0.001; ‘Long pre‐hospital duration’ OR = 0.69 (0.37–1.31), *p* = 0.26; ‘Has diagnosis of epilepsy’ OR = 2.19 (1.04–4.61), *p* = 0.04; ‘Admitted to intensive care’ OR = 1.53 (0.81–2.89), *p* = 0.19; ‘Infection‐related’ OR = 0.22 (0.10–0.47), *p* < 0.001. Abbreviations: AUC, area under the curve; LASSO, least absolute shrinkage and selection operator; ROC, receiver operating characteristic.

Overall mortality in our cohort was 9.4%, with 4.9% occurring in the first year following the status epilepticus (Figure 4a). To identify patients with a higher risk of mortality, we used a LASSO regression model, which selected six clinical variables significantly associated with long‐term mortality. This model demonstrated good predictive accuracy (area under the curve = 0.78; Figure 4b). Among these variables, three were independently statistically significant: progressive semiology and previous diagnosis of epilepsy were associated with increased mortality, while infection‐related status epilepticus was associated with reduced mortality (Figure 4c).

## DISCUSSION

In this paper, we summarize data from more than 400 patients who presented with paediatric status epilepticus at a tertiary paediatric hospital in Switzerland over a 12‐year period. To our knowledge, this is the largest contemporary Swiss paediatric cohort of status epilepticus. Our findings highlight the heterogeneity of status epilepticus in children, with acute infectious and parainfectious aetiologies playing a major role in early childhood, while status epilepticus associated with an established epilepsy diagnosis accounted for a greater proportion of cases in older children. Our data also validate previous work showing that time to first intervention is a key determinant of immediate morbidity and treatment course of patients. We further found that status epilepticus‐related mortality was more likely in patients with progressive aetiologies or pre‐existing epilepsy and less likely in those with infectious aetiologies.

In the canton of Zurich there are three hospitals that are able to attend to paediatric medical emergencies where patients can present.^29^ Most cases of paediatric status epilepticus, however, are managed at the University Children's Hospital Zurich, the region's primary referral centre. According to the latest statistics,^30^ the canton of Zurich has a population of approximately 1 539 275, of whom 19.8% are younger than 19 years old. On the basis of our cohort of 467 patients, we estimate an incidence of status epilepticus in the canton of Zurich of 15.3 per 100 000 children and adolescents per year. We expect our centre to receive the majority of patients presenting with status epilepticus. Therefore, our estimates of incidence should largely reflect those for the canton at large. This incidence is consistent with reported rates from other high‐income countries such as in the USA (20 per 100 000)^10^ and Germany (17.6 per 100 000)^1^ and the French‐speaking part of Switzerland (21 per 100 000).^31^ This consistency suggests that our data set captures a representative proportion of cases of paediatric status epilepticus in the region. Our observed mortality rate of 9.4% was higher than the 3% reported in a similar German study.^10^ However, this discrepancy probably reflects differences in study methodology, as that study only considered in‐hospital mortality, whereas our study includes longer‐term outcomes. Notably, our 30‐day mortality rate was 1.5%.

Using cluster analysis, we identified three main subgroups of patients: (1) a ‘febrile seizure’ cluster, primarily involving younger children with status epilepticus triggered by infection, (2) the ‘severe parainfectious’ cluster, characterized by refractory status epilepticus often associated with infections, and (3) the ‘known epilepsy’ cluster, comprising patients with established epilepsy diagnoses and a broader age range. The younger children in the first two clusters were difficult to distinguish at presentation, emphasizing the need for biomarkers to predict which cases of febrile or infectious status epilepticus may require intensive care. The ‘severe parainfectious’ status epilepticus group emerges as a distinct cluster, once treatment variables are included. This cluster therefore reflects a differential response to treatment, which itself may be caused by different aetiologies compared, such as more severe infections and distinct genetic susceptibilities, than the patients who remain in the ‘febrile seizures’ cluster, the evaluation of which is outside the scope of this analysis. These clusters revealed important management trends, such as the increased likelihood of patients in the ‘known epilepsy’ and ‘severe parainfectious’ clusters requiring escalation beyond first‐line treatment. This finding corroborates previous efforts in adult status epilepticus to identify key clinical features for characterizing patients’ profiles,^32^ guiding management,^33^ and developing prognostic scoring systems.^34^ Furthermore, more than half of the episodes of status epilepticus in our cohort required escalation to at least second‐line treatment (57%) and ICU admission (51%). These figures probably reflect the referral patterns of our institution, which serves as the final centre for cases that cannot be managed at less specialized facilities, rather than indicating the inherent severity of status epilepticus in the region. Robustness was highest at admission and decreased as additional post‐admission variables were incorporated, consistent with the curse of dimensionality whereby increasing the number of variables can reduce the stability of distance‐based clustering solutions. Using all available data, robustness was 69.1%, indicating moderate reproducibility of cluster assignments.

We also evaluated clinical variables associated with long‐term mortality in paediatric status epilepticus. Patients with a previous diagnosis of epilepsy, particularly those with progressive aetiologies, were at the highest risk of mortality, while infectious aetiologies were associated with a lower mortality risk. LASSO regression revealed additional predictive factors, including non‐convulsive semiology and longer duration of pre‐hospital status epilepticus, associated with a decreased mortality risk, and ICU admission, associated with an increased mortality risk. While these predictors were not independently significant, they are consistent with previous findings.^35^

This study is limited by its retrospective nature, with data retrospectively collected from clinical notes, emergency department codes, and EEG reports from a single centre spanning a 12‐year period. Moreover, the analyses reported quantify unadjusted associations and may therefore be influenced by clinical confounders such as age, underlying aetiology, and treatment location. While these findings may have regional relevance, there are limitations to their generalizability, specifically how well our data‐driven cluster summary applies to cohorts of patients in other settings. In addition, real‐world clinical data sets are often incomplete. The pragmatic approach used here of treating ‘unknown’ values as a separate category may unintentionally capture patterns related to data missingness rather than underlying pathophysiology. We cannot exclude the possibility that missingness itself reflects illness severity, treatment urgency, or documentation practices, and may therefore be associated with treatment response or mortality. In future, generalizability across cohorts could be assessed by evaluating how well our clustering separates clinical groups in an unrelated data set through silhouette score,^36^ but this is outside the scope of the current analysis. Additionally, changes in practice may change reproducibility of clinical clusters over time. For example, certain diagnostic categories such as autoimmune or genetic causes increase over time with improved yield of advancing diagnostics. Similarly changes in the indication for EEG over time may affect some findings. Furthermore, mortality accounted for 9.4% of admissions, resulting in class imbalance in the logistic regression. This may reduce sensitivity to mortality and could potentially be addressed by using weighted logistic regression or up‐sampling the minority class.

Given the role of the University Children's Hospital Zurich as the region's primary referral centre, this data set probably captures most cases of paediatric status epilepticus outside the neonatal period in the region. The 12‐year study span introduces potential variability due to evolving etiological understanding, particularly advances in autoimmune^37^, ^38^ and genetic diagnostics,^39^ which may have affected the distribution of status epilepticus aetiologies over time. Additionally, we did not assess benzodiazepine dosing during first‐line treatment, a known factor contributing to poor response.^33^, ^40^ NCSE was included in the analysis but may be under‐recognized, introducing potential bias. However, semiology had a limited influence on the clustering (Figure 3c). Furthermore, NCSE differs from convulsive status epilepticus significantly in terms of clinical recognition, typical pathways of patients, and likely underlying pathophysiology. We have deliberately included cases with NCSE in our collection to reflect clinical reality, but cases with NCSE did not drive any of the observed clinical clusters (Figure S1). Another important limitation is the onset time of status epilepticus, especially for patients where status epilepticus starts out‐of‐hospital, dependent on collateral history by a bystander or healthcare provider witnessing the event. Therefore, the actual onset time of status epilepticus and the subsequent measures of latency to admission and treatment need to be interpreted in view of this inherent inaccuracy.

Despite these limitations, this study, the largest from Switzerland, provides novel insights into the presentation and management of paediatric status epilepticus and contributes to the growing body of clinical data from high‐income countries. The use of machine learning to identify clinical patterns offers a novel approach to characterizing status epilepticus subgroups, offering a foundation for further research. We hope these findings inspire additional prospective studies to refine understanding and improve outcomes for children with status epilepticus.

## CONFLICT OF INTEREST STATEMENT

The authors have stated that they had no interests that might be perceived as posing a conflict or bias.

## Supporting information


**Figure S1:** Cluster analysis highlighting patients who presented with non‐convulsive status epilepticus.

## Data Availability

De‐identified anonymized clinical data used in this study are available along with analysis code from the corresponding author upon reasonable request.
